# The human RAP1 and GFAPɛ proteins increase γ-secretase activity in a yeast model system

**DOI:** 10.1093/g3journal/jkad057

**Published:** 2023-03-17

**Authors:** Mark J Swanson, Kelsey N Lewis, Robert Carpenter, Alexis Whetzel, Nancy S Bae

**Affiliations:** Department of Biochemistry and Molecular Genetics, Midwestern University, Glendale, AZ 85308, USA; Department of Biochemistry and Molecular Genetics, Midwestern University, Glendale, AZ 85308, USA; Department of Biomedical Sciences, College of Graduate Studies, Midwestern University, Glendale, AZ 85308, USA; Department of Biochemistry and Molecular Genetics, Midwestern University, Glendale, AZ 85308, USA; Department of Biochemistry and Molecular Genetics, Midwestern University, Glendale, AZ 85308, USA

**Keywords:** *Saccharomyces cerevisiae*, RAP1, TERF2IP, GFAPɛ, γ-secretase, Alzheimer's disease, amyloid beta

## Abstract

Alzheimer's disease (AD) is an age-related disorder that results in progressive cognitive impairment and memory loss. Deposition of amyloid β (Aβ) peptides in senile plaques is a hallmark of AD. γ-secretase produces Aβ peptides, mostly as the soluble Aβ40 with fewer insoluble Aβ42 peptides. Rare, early-onset AD (EOAD) occurs in individuals under 60 years of age. Most EOAD cases are due to unknown genetic causes, but a subset is due to mutations in the genes encoding the amyloid precursor protein that is processed into Aβ peptides or the presenilins (PS1 and PS2) that process APP. PS1 interacts with the epsilon isoform of glial fibrillary acidic protein (GFAPɛ), a protein found in the subventricular zone of the brain. We have found that GFAPɛ interacts with the telomere protection factor RAP1 (TERF2IP). RAP1 can also interact with PS1 alone or with GFAPɛ in vitro. Our data show that the nuclear protein RAP1 has an extratelomeric role in the cytoplasm through its interactions with GFAPɛ and PS1. GFAPɛ coprecipitated with RAP1 from human cell extracts. RAP1, GFAPɛ, and PS1 all colocalized in human SH-SY5Y cells. Using a genetic model of the γ-secretase complex in *Saccharomyces cerevisiae*, RAP1 increased γ-secretase activity, and this was potentiated by GFAPɛ. Our studies are the first to connect RAP1 with an age-related disorder.

## Introduction

Alzheimer's disease (AD) is an insidious, age-related disorder that results in progressive, irreversible cognitive impairment and memory loss, affecting millions of elderly people worldwide. The deposition of amyloid peptides in senile plaques is a hallmark of AD. Though most cases are late-onset AD, occurring in individuals in their 60’s, 1–5% of cases occur in younger people (Bekris *et al.* 2010; Zhu *et al.* 2015). Early-onset AD (EOAD) afflicts individuals from their 30’s to 60’s; 10–15% of these are due to mutations in one of three genes: *APP*, *PSEN1*, or *PSEN2* (Jarmolowicz *et al.* 2015). The *APP* gene encodes the amyloid precursor protein, which has a single transmembrane domain embedded in the plasma membrane of the cell (Zhang *et al.* 2011). β-Secretase is an enzyme that cleaves APP, producing a fragment of the protein made up of the carboxyl(C)-terminal 99 amino acids, called C99, that is embedded in the plasma membrane. γ-Secretase is a multi-subunit protein complex. It contains a catalytic subunit, either presenilin 1 or 2 proteins (PS1 or PS2, encoded by the *PSEN1* and *PSEN2* genes, respectively) (Kimberly *et al.* 2003). Mutations in *PSEN1* are more commonly the cause of EOAD than mutations in *PSEN2* or *APP* (Cruts *et al.* 2012). Three non-enzymatic subunits are required for activity: anterior pharynx-1 (Aph-1), nicastrin (Nic), and presenilin enhancer 2 (Pen2 or PSENEN). γ-Secretase cleaves C99 within the membrane spanning portion at one of several places, resulting in two products. The amino(N)-terminal fragments are Aβ peptides that are released outside of the cell. The C-terminus forms the amyloid precursor protein intracellular domain (AICD), which is released from the plasma membrane and enters the nucleus where it affects gene expression (Multhaup *et al.* 2015). Most Aβ peptides produced are 40 amino acids in length (Aβ40) and are soluble (Zhang *et al.* 2011). The less soluble Aβ42 peptides are less frequently made. When the ratio of Aβ40:42 favors the more soluble Aβ40, the brain functions without AD-related pathology. Higher levels of Aβ42 promote self-aggregation resulting in the formation of senile plaques. Mutations that are associated with EOAD exert their effects by either increasing the overall amount of Aβ peptides produced or by increasing the ratio of Aβ42 to Aβ40 (Weggen and Beher 2012).

Among the proteins interacting with γ-secretase is an isoform of the glial fibrillary acidic protein (GFAP). GFAP is an essential intermediate filament protein predominantly expressed by astrocytes within the brain. There are at least 10 different GFAP isoforms, and astrocytes preferentially express different isoforms of GFAP in different areas of the brain. GFAPα (isoform 1) is 432 amino acids in length, and it is the predominant isoform capable of forming filaments (Yang and Wang 2015). One minor isoform, GFAPɛ (isoform 3), results from a combination of alternative splicing and polyadenylation to produce a 431 amino acid protein that is identical to GFAPα except in the C-terminus of the protein, amino acids 391–431 (Nielsen *et al.* 2002). GFAPɛ is expressed by neurogenic astrocytes in the subventricular zone of the brain, a site of adult neurogenesis (Roelofs *et al.* 2005). Studies have also shown increased GFAPɛ expression in reactive astrocytes near amyloid beta plaques, indicating a possible role for GFAPɛ in AD pathology (Kamphuis *et al*. 2014). GFAPɛ was found to interact with the *N*-terminal, cytoplasmic domains of PS1 (amino acids 1–85) and PS2 (amino acids 1–93), and the interactions required the C-terminal half of GFAPɛ (amino acids 204–431), including the ɛ-specific sequences (Nielsen *et al.* 2002). PS1 did not interact with GFAPα.

Aging at the cellular level is associated with telomeres, which are tandem repeat sequences found at the ends of eukaryotic chromosomes (Blackburn *et al.* 2015) that stabilize and protect chromosomes from end-fusion. As cells progress through successive cell cycles, DNA polymerases are incapable of replicating chromosome ends, so they become shorter over time, leading to replicative senescence before critical chromosomal information is compromised (Shay and Wright 2000). When cells are not actively replicating, a six-membered protein complex called shelterin protects telomeres from fusion and degradation (Palm and de Lange 2008). The telomeric repeat factor 1 (TRF1) and 2 (TRF2) proteins bind directly to the double-stranded telomeric repeats, while protection of telomeres protein 1 (POT1) binds to the single-stranded DNA overhang that occurs at the end of each telomere. TRF2-interacting nuclear protein 2 (TIN2) and tripeptidyl peptidase 1 (TPP1) connect TRF1 and TRF2 with POT1. The final subunit, RAP1 (repressor activator protein 1, a.k.a. TERF2IP), is recruited to telomeres by TRF2 and is responsible for protecting telomeres from illegitimate recombination and thus maintains genome stability (Bae and Baumann 2007). We have found that as human dermal fibroblasts undergo successive rounds of replication, their telomeres decreased in length, and TRF2 levels decreased concomitantly (Swanson *et al.* 2016). However, RAP1 levels remained more stable as cells progressed through cell divisions, suggesting that RAP1 may play additional roles in aging cells. To identify novel functions of the human RAP1 protein, we utilized a yeast two-hybrid (Y2H) screen and discovered that human GFAPɛ interacts with RAP1. We show that RAP1 interacts with GFAPɛ but not with GFAPα. We also show that RAP1, GFAPɛ, and the N-terminal domain of PS1 all interact in vitro, likely forming a ternary complex. This is further supported by the fact that all three proteins colocalize in the cytoplasm of SH-SY5Y neuroblastoma cells. Using a reconstituted γ-secretase system in the yeast *Saccharomyces cerevisiae*, we show that RAP1 and GFAPɛ can increase γ-secretase activity. Altogether, our data implicate a new role for human RAP1 in an age-related disease.

## Materials and methods

### Yeast strains and media

Yeast strains used in this study are listed in Supplementary Table 1. Yeast rich media (YPAD) and selective synthetic complete (SC) drop-out media supplemented as noted were prepared as described (Adams *et al.* 1997). The *kanMX6* marker was selected on YPAD agar medium with 300 μg/mL G418 sulfate. To select against the wild-type *URA3* gene, 5-fluoroorotic acid (5FOA) was added to SC media at 1 g/L. To select against the *HIS3* gene product, 3-Amino-1,2,4-triazole (3AT) was added to SC media at 10 mM. X-gal and X-α-gal were added to SC media at a final concentration of 0.04 mg/mL. Yeast cells were transformed with plasmids or linear DNA as described (Gietz and Schiestl 2007).

### Cloning and plasmids

Standard recombinant DNA techniques were used for the construction of plasmids (Sambrook and Russell 2001). Cloning details for plasmids created in this study are described in the supplementary materials and methods. Primers used in amplification of DNA for cloning are listed in Supplementary Table 2.

### Yeast two-hybrid screening and analysis

The Y2H screen was performed using the ProQuest Two-Hybrid System (Invitrogen). The full-length human *RAP1* cloned in frame with the GAL4 DNA binding domain (GAL4_DBD_) of vector pDBLeu (pDBLeu/RAP1) was used as the bait, and a human fetal brain cDNA library in plasmid pPC86 was used as the prey. Transformed MaV203 cells (Supplementary Table 1; Vidal *et al*. 1996) were selected on SC-leu-trp plates then replica printed to SC-leu-trp-his plates with 3AT to select for potential interactors. 3AT resistant transformants that exhibited blue color on SC-leu-trp plates with X-gal were isolated. Library plasmids were rescued in *Escherichia coli* DH5α. Each was retested in MaV203 with pDBLeu/RAP1 to confirm the interaction, and the inserts were analyzed by sequencing.

**Fig. 1.**
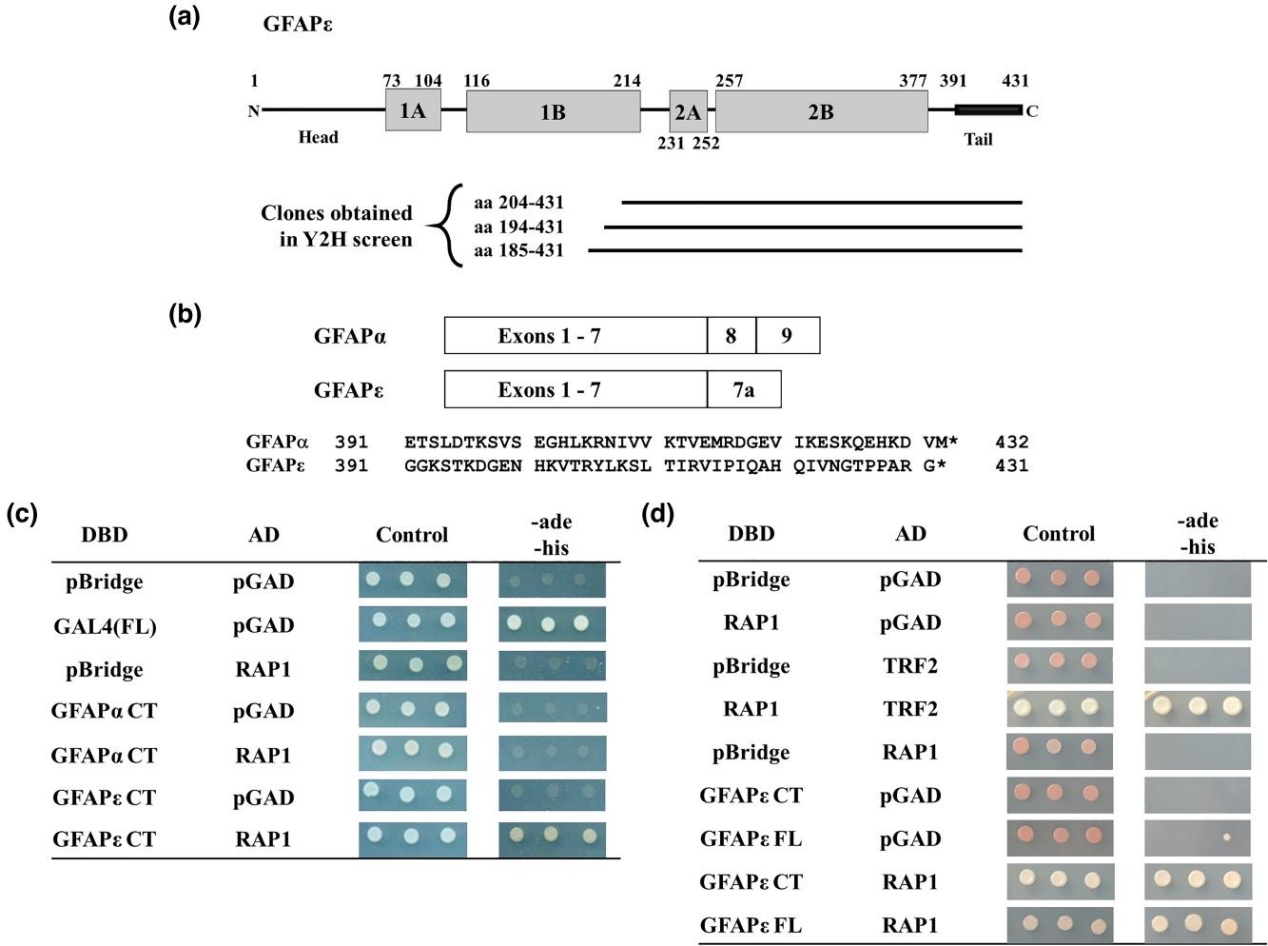
Human RAP1 interacts specifically with the epsilon isoform of glial fibrillary acidic protein (GFAPɛ) in the Y2H system. a) The domain organization of the GFAPɛ protein is shown at the top of the image. GFAPɛ consists of α-helical rod-domains (grey boxes) flanked by a head and tail domain. The thick portion of the tail domain is the segment unique to the GFAPɛ isoform. Below the diagram are lines depicting the segments of GFAPɛ that were isolated as Gal4p activation domain fusions from the Y2H library. b) GFAPα and GFAPɛ are compared. The boxes at the top depict the exons included for the differentially spliced forms, which encode distinct tail regions. Below is a comparison of the protein sequence differences between the isoforms. The isoforms are identical in the N-terminal 390 amino acids but differ in their C-termini (42 amino acids for GFAPα and 41 amino acids for GFAPɛ). c) Y2H Gold cells transformed with plasmids show an interaction between RAP1 and GFAPɛ but not with GFAPα. Three biological replicates (independent transformants) of each were grown in liquid SC-trp-leu medium overnight before being diluted 1:10 and spotted onto SC-trp-leu (control) medium and SC-trp-leu-ade-his medium. GAL4_DBD_ fusions are shown in the first column, and GAL4_AD_ fusions are shown in the second column. The GFAP fusions used are based on the smallest fragment isolated in the Y2H screen, GFAPα 204–432 and GFAPɛ 204–431. d) Y2H Gold cells were transformed with RAP1 and the full-length GFAPɛ protein. The experiment was set up as in (c), except GFAPɛ 204–431 and GFAPɛ 1–431 were tested for interaction with RAP1.

Interactions were retested using the Matchmaker Gold yeast two-hybrid system (Takara Bio, USA). Plasmids were transformed into Y2H Gold cells (Supplementary Table 1). Phenotype tests for determining interactions were performed using three biological replicates (independent transformants) for each combination of plasmids. Cells were grown to saturation at 30°C in a 24-well plate in SC-leu-trp medium with shaking. Serial dilutions (1:10) were made and plated onto media as indicated, incubated for 3 days at 30°C then photographed. The images in Fig. 1c and d are from the same plates, but the images were cropped to remove redundant or irrelevant samples and to organize the images better.

### Yeast gamma secretase reporter strain construction

The *URA3::MEL1*_UAS_*–MEL1*_TATA_*-AUR1-C* reporter gene was eliminated from Y2H Gold to allow the use of the *URA3* marker on a plasmid. Y2H Gold cells were plated onto SC plates containing 5FOA, incubated at 30°C for 3 days, and colonies were streaked onto fresh SC + 5FOA plates. Loss of the *URA3* gene function in the resulting strain (MSY4: Y2H Gold 5FOA^R^) was confirmed by lack of growth on SC-ura plates and by complementation with a *URA3*-containing plasmid.

A gene expressing a SUC2-C99-GAL4 fusion protein was marked with *kanMX6* and flanked by sequences to either side of the *MET17* gene to create Δ*met17::SUC2-C99-GAL4::kanMX6*. Linearized DNA was transformed into the MSY4 yeast strain, selecting on YPAD agar medium with G418 sulfate, and verified by PCR to ensure the *C99-GAL4* fusion was inserted into the chromosome, replacing *MET17,* creating strain MY14.

### Yeast gamma secretase activity

We expressed the four subunits of γ-secretase and RAP1 and GFAPɛ, individually or together, in *S. cerevisiae* from plasmids containing bidirectional promoters (pBEVY plasmids; Miller *et al.* 1998). Spotting assays were conducted as described above for the Y2H retest. Quantitative α-galactosidase assays were performed as described in the Yeast Protocols Handbook for the Matchmaker Gold yeast two-hybrid system (Takara Bio, USA).

### Interactions using *E. coli* expressed proteins

Proteins with the affinity tags 6 × histidine (His_6_), glutathione S-transferase (GST), and maltose binding protein (MBP) fused to their amino termini were expressed in and purified from *E. coli* as described (Greenfield *et al.* 2020) with the following modifications. GST- and MBP-tagged proteins were expressed in BL21 or HMS174(DE3) cells, and His_6_-tagged proteins were expressed in BL21(DE3) or HMS174(DE3) cells. The cultures were induced for 2–3 h at 37°C or for 16–18 h at 18°C (GFAP proteins). Cell pellets were resuspended in lysis/binding buffer (50 mM Tris pH7.5, 200 mM NaCl, 10–20 mM β-mercaptoethanol, 1 × Problock protease inhibitors (Gold Biotechnology; GB-116–10)). For cells expressing GFAPα or GFAPɛ, NP-40 was added to a final concentration of 1%. The samples were sonicated before using them in pulldown experiments. Protino Glutathione Agarose 4B (Machery-Nagel GmbH & Co.) was used for binding GST-tagged proteins. A 10-mM glutathione solution was used to elute proteins from the beads. Purification of MBP-tagged proteins was performed using Amylose Resin (New England Biolabs, E8021S), according to the manufacturer's protocol. His_6_-tag proteins were isolated on His-Bind Resin (Novagen, #69670) according to the manufacturer's protocol and eluted with 300 mM imidazole. For interactions, eluted purified proteins or cell lysates were added to proteins bound to beads. Before adding the His_6_-Rap1, His_6_-GFAPɛ, His_6_-GFAPα, or MBP-PS1 for coprecipitation experiments, the elution buffer was exchanged using a 10-kDa filter column via centrifugation.

### Human cell growth, transfection, and enzyme-linked immunosorbent assays

The human neuroblastoma cell line SH-SY5Y and the human glioblastoma cell line U251 were propagated in Dulbecco's Modified Eagle Medium supplemented with 10% fetal bovine serum and non-essential amino acids at 37°C in a 5% CO_2_ environment. For co-immunoprecipitations, cells were harvested and lysed in InterPlay TAP Purification Kit Lysis Buffer (Agilent, 240107-51) with protease inhibitors (ThermoFisher Scientific, PIA32955). Protein concentrations were measured using the Pierce BCA Protein Assay Kit (ThermoFisher Scientific, PI23225).

Transfection was accomplished using ViaFect Transfection Reagent (Promega, E4982) according to the manufacturer's guidelines. For microscopy, a sterile cover slip was placed inside a six-well plate, and cells were plated into the well. When cells were harvested 48 h post transfection, coverslips were processed for immunofluorescent work, and the remaining cells in the well were harvested with SDS sample loading buffer for immunoblotting (described below). For enzyme-linked immunosorbent assays (ELISAs), equal numbers of cells were seeded into six-well plates. Six wells were transfected with pLPC vector, and six wells were transfected with pLPC-hRAP1 FL. Medium was harvested 48 h post transfection, and Aβ40 and Aβ42 levels were analyzed using Amyloid beta 40 Human ELISA Kit (Invitrogen, KHB3481) and Amyloid beta 42 Human ELISA Kit (Invitrogen, KHB3441) according to the manufacturer's instructions.

### Co-immunoprecipitation

Protein G Dynabeads (Invitrogen) were used according to the manufacturer's protocol. To precipitate RAP1, rabbit monoclonal TERF2IP (D9H4) antibodies were used (Cell Signaling Technology, 5433S). As a control, a non-specific Rabbit IgG antibody (Cell Signaling Technology, 2729S) was used. The antibody-bound beads were crosslinked using bis(sulfosuccinimidyl) suberate (BS3; ThermoFisher Scientific, PI21580) according to the manufacturer's protocol. Human cell lysates were added to the crosslinked beads and incubated at 4°C. The proteins were eluted with 0.1-M Glycine pH 2.8. The pH of the eluate was adjusted by adding an equal volume of 1-M Tris-HCl pH 7.5.

### Immunoblotting

Immunoblotting was performed as described (Mahmood and Yang 2012). Proteins were resolved using 10% SDS-PAGE and transferred to nitrocellulose membranes. Briefly, membranes were blocked with 5% non-fat dry milk (NFDM) in 1 × Tris-buffered saline/0.1% Tween-20 (TBST), incubated with primary antibodies in 5% NFDM/TBST, followed by incubation with secondary antibodies in 1% NFDM/TBST. The following primary antibodies were used to detect the affinity tags on the *E. coli* expressed proteins: rabbit polyclonal anti-His antibodies H-15 (Santa Cruz Biotechnology, sc-803); mouse monoclonal anti-His antibodies (Millipore, 05–949); rabbit polyclonal anti-GST antibodies (ThermoFisher Scientific, PI700775); mouse monoclonal anti-GST antibodies (26H1) (Cell Signaling Technologies, 2624); anti-MBP antibodies (New England Biolabs, E8032). For secondary antibodies, goat anti-mouse and goat anti-rabbit conjugated to IRDye 680RD (LI-COR Biosciences, 926-68170 and 926-68171, respectively) and goat anti-mouse and goat anti-rabbit conjugated to IRDye 800CW (LI-COR Biosciences, 827-08364 and 827-08365, respectively) were used. Detection was done using a LI-COR Odyssey CLX Imager.

Immunoblotting for human proteins was done using the following antibodies: rabbit monoclonal TERF2IP (D9H4) antibodies (Cell Signaling Technology, 5433S); rabbit polyclonal anti-GFAP δ antibodies (EMD Millipore, AB9598); and HRP-conjugated goat anti-rabbit antibodies (ThermoFisher Scientific). The membranes were incubated with ECL Prime Western Blotting Detection Reagent (ThermoFisher, 45-002-401) before imaging using a Bio-Rad ChemiDoc XRS + instrument.

### Microscopy

Cells were plated and grown overnight in a six-well plate with 22 × 22 mm glass coverslips in each well. Cells were fixed using Histochoice MB Fixative (Electron Microscopy Science) then blocked at room temperature with 5% donor horse serum in 1 × Phosphate-buffered saline/0.1% Tween-20 (PBST). The samples were first incubated with primary antibodies to GFAPɛ (described above) or PSENEN (rabbit polyclonal anti-PEN2 antibodies, Invitrogen, PA5-20302) in blocking solution. For secondary antibodies, goat anti-rabbit IgG (H + L) antibodies conjugated to Alexa Fluor 488 (Invitrogen, A32731) were used. The samples were washed with 1 × PBS before adding additional primary antibodies. Cells were blocked again then incubated with rabbit polyclonal anti-TERF2IP antibody conjugated to Alexa Fluor 594 (Novus Biologicals, NB100-292AF594) or with rabbit polyclonal anti-Presinilin-1 antibody conjugated to Alexa Fluor 405 (Novus Biologicals, NBP1-76792AF405) when FLAG-RAP1 was expressed in the cells. The samples were washed with 1 × PBS. To detect FLAG-RAP1, a third primary antibody was added, mouse monoclonal anti-DYKDDDDK (9A3) Tag antibodies (Cell Signaling Technology, 8146S), which was detected with goat anti-mouse antibodies conjugated to Alexa Fluor 594 (Invitrogen, A32742). Coverslips were mounted on slides using DAPI Fluoromount-G (Southern Biotech), or Fluoromount-G when the Presenilin-1 antibodies were included. Fluorescence microscopy was carried out using an ECHO Revolve microscope (VWR, USA). Fluorescence images were obtained using a 60 × oil-immersion lens. Z-stacks were acquired with a 0.3-μm Z step size and composite images generated using the Extended Depth of Focus setting in the ECHO Pro software.

### Statistical analyses

For the α-galactosidase assays, three or more biological replicates (independent transformants) were used. In Fig. 5b, one technical replicate for each of four biological replicates were compared for each sample type. Statistical analysis was done with Microsoft Excel software using a one-tailed student's t-test since we expected the activity to increase when the γ-secretase subunits were expressed. In Fig. 5c, three technical replicates for each of three biological replicates were assayed for each sample type, and the technical replicates were averaged to determine the value of each biological replicate. Statistical analysis was done using GraphPad Prism software using a nested, one-way ANOVA with a Tukey's multiple comparisons test. In Fig. 5d, two technical replicates for each of six transfected wells of cells were assayed for vector and RAP1 overexpressing cells. The technical replicates were averaged to determine the value of each biological replicate. Statistical analysis was done with Microsoft Excel software using a two-tailed student's t-test. Each bar graph displays the means of the replicates with error bars that represent the standard error of the mean of the biological replicates.

## Results

### The human RAP1 protein interacts with the epsilon isoform of GFAP in the yeast two-hybrid system

To identify novel, non-telomeric roles for the human RAP1 protein, we performed a yeast two-hybrid (Y2H) screen to isolate previously unknown interacting proteins. Full-length, human RAP1 protein fused to the Gal4p DNA binding domain (GAL4_DBD_) was used as bait to screen a human fetal brain cDNA library (prey) fused to the Gal4p activation domain (GAL4_AD_). Approximately 610,000 transformants of MaV203 (Supplementary Table 1) were screened, and those that were able to grow on 3AT-containing medium and displayed a blue color on plates with X-gal were further studied. Library plasmids that did not activate the reporter genes without the bait were isolated and retested with GAL4_DBD_-RAP1 to confirm the interaction. Three of the cDNAs encoded varying length fragments of the carboxyl-terminus of the epsilon isoform of the GFAP protein (GFAPɛ; Fig. 1a). The alpha isoform is the predominant form of GFAP in human cells; GFAPα and GFAPɛ differ only in their carboxy-terminal 42 or 41 residues, respectively (Fig. 1b). We retested the interaction of RAP1 with the smallest GFAPɛ fragment obtained from the Y2H screen (amino acids 204–431) and compared it to the corresponding GFAPα fragment (204–432). GAL4_DBD_ vector with GAL4_AD_-RAP1 fusion, GAL4_DBD_-GFAPα CT fusion with GAL4_AD_ vector, and GAL4_DBD_-GFAPɛ CT fusion with GAL4_AD_ vector did not grow on SC-leu-trp-his-ade medium, showing individual fusion proteins did not activate the reporter genes.

The only combination that enabled growth on SC-leu-trp-his-ade medium was GAL4_DBD_-GFAPɛ CT with GAL4_AD_-RAP1, indicating that the interaction with RAP1 is specific for the GFAPɛ isoform. Unlike Nielsen *et al*. (2002), we did not isolate a cDNA encoding the full-length GFAPɛ. Since GFAPɛ is not known to exist in human cells as a carboxy-terminal fragment, we tested the full-length protein for interaction with RAP1. Cells expressing GAL4_DBD_-GFAPɛ carboxy-terminal (CT) or full-length (FL) fusion proteins with the GAL4_AD_-RAP1 were able to grow on the SC-leu-trp-his-ade medium. Thus, RAP1 interacts specifically with the GFAPɛ isoform, and the interaction is localized to the carboxy-terminal half of GFAPɛ (amino acids 204–431) that contains the GFAPɛ specific domain.

### Bacterially expressed RAP1, GFAPɛ, and PS1 directly interact with one another

**Fig. 2.**
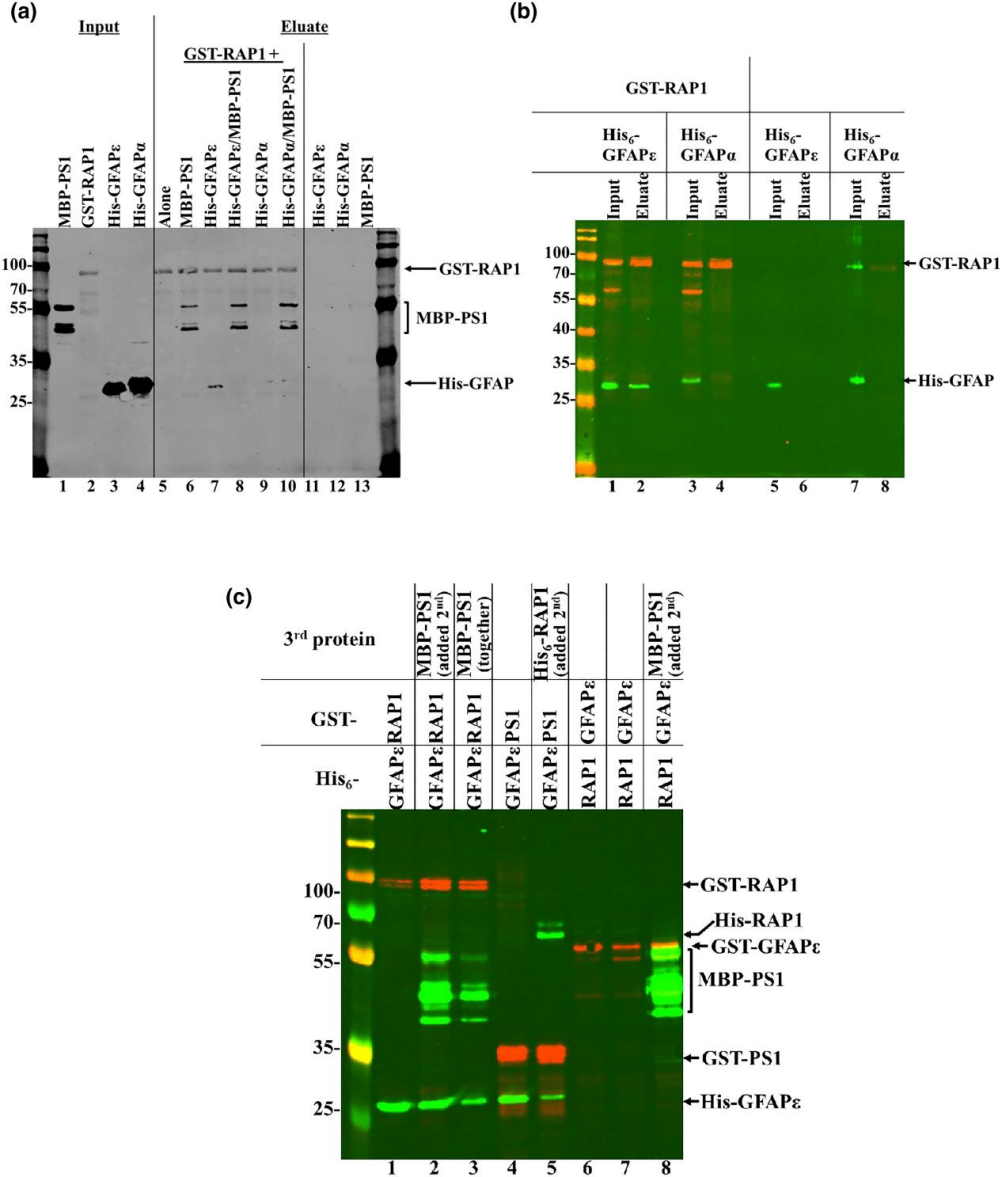
Human RAP1, GFAPɛ and PS1 interact with one another in vitro. a) Proteins were expressed in BL21 and BL21(DE3) cells. MBP-PS1_1–85_ and the His_6_-tagged GFAP proteins (amino acids 204—end) were purified prior to being added to glutathione beads alone or beads prebound with GST-RAP1. The input lanes show the purified proteins (lanes 1, 3, and 4) or GST-RAP1 lysate (lane 2). Eluates from the glutathione beads are in lanes 5 through 13. Proteins included in each sample are listed above the blot image. The blot was probed simultaneously with primary antibodies to each of the tags, and IRDye secondary antibodies were used for detection. The protein bands are labeled to the right of the blot image. b) His_6_-tagged GFAP proteins (amino acids 204—end) were expressed together with GST-RAP1 (lanes 1 through 4) or individually (lanes 5 through 8). Lysates (inputs) were mixed with glutathione beads, and eluted proteins were resolved on the gel. The blot was simultaneously probed with antibodies to GST and His_6_, and IRDye secondary antibodies were used for detection. The protein bands are labeled to the right of the blot image. c) The GST and His_6_-tagged proteins were expressed together in HMS174(DE3) cells. Where indicated, a third protein expressed separately in HMS174(DE3) cells was added. Only eluates are shown for simplicity. The blot was simultaneously probed with antibodies to GST, His_6_, and MBP. IRDye secondary antibodies were used for detection. The protein bands are labeled to the right of the blot image.

GFAPɛ has been reported to interact with the catalytic subunit of γ-secretase complex, PS1 (Nielsen *et al*. 2002). We tested for direct interactions among RAP1, PS1, and GFAPɛ using affinity-tagged proteins expressed in *E. coli*. His_6_-GFAPɛ coprecipitated with GST-RAP1, but His_6_-GFAPα did not (Fig. 2a, lanes 7 and 9, respectively) confirming the results from the Y2H assays showing the interaction with RAP1 is specific for GFAPɛ. MBP-PS1_1–85_ also coprecipitated with GST-RAP1, indicating a direct interaction (Fig. 2a, lane 6). When GST-RAP1 was incubated with either of the His_6_-GFAP proteins and MBP-PS1_1–85_, only MBP-PS1_1–85_ coprecipitated (Fig. 2a, lanes 8 and 10). One issue with the purifications was the insolubility of the GFAP proteins (data not shown), which was also described by Nielsen *et al.* (2002). When we switched to HMS174(DE3) *E. coli* cells and lysed them with 1% NP-40, which solubilized the GFAP proteins, His_6_-GFAPɛ coprecipitated with GST-RAP1 (Fig. 2b, lane 2), but His_6_-GFAPα did not coprecipitate with RAP1 (Fig. 2b, lane 4).

We retested the interactions switching some of the tags on the proteins and utilizing co-expression in HMS174(DE3) cells. The results in Fig. 2c show that His_6_-GFAPɛ coprecipitated with GST-RAP1 (Fig. 2c, lane 1), and when MBP-PS1_1–85_ was added to the pre-bound GST-RAP1/His_6_-GFAPɛ or simultaneously with them, all three proteins coprecipitated (Fig. 2c, lanes 2 and 3). His_6_-GFAPɛ also coprecipitated with GST-PS1_1–85_ (Fig. 2c, lane 4), and when His_6_-RAP1 was added to the prebound GST-PS1_1–85_/His_6_-GFAPɛ, all three proteins coprecipitated (Fig. 2c, lane 5). MBP-PS1_1–85_ coprecipitated with GST-GFAPɛ (Fig. 2c, lane 8). However, His_6_-RAP1 was not able to coprecipitate with GST-GFAPɛ (lane 6) even when MBP-PS1_1–85_ was included (Fig. 2c, lane 7). These data indicate that RAP1, GFAP ɛ, and PS1_1–85_ can form a complex in vitro.

### GFAPɛ co-immunoprecipitates with RAP1 from sh-SY5Y neuroblastoma cells

**Fig. 3.**
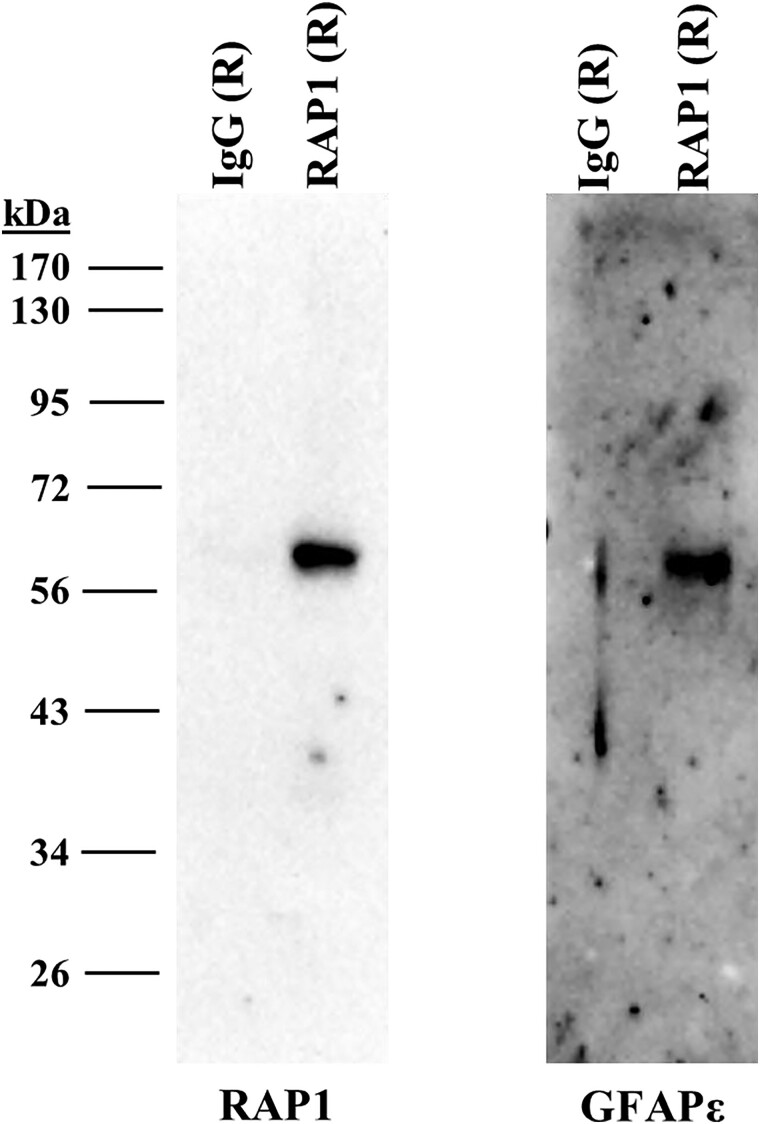
GFAPɛ coimmunoprecipitates with RAP1 from human SH-SY5Y neuroblastoma cells. SH-SY5Y cells were grown and lysed. Rabbit antibodies to RAP1 or non-specific rabbit antibodies were used to precipitate proteins that were eluted and resolved by SDS-PAGE. The panel on the left shows a membrane probed with the antibodies to RAP1. The panel on the right shows an identically loaded membrane probed with antibodies to GFAPɛ.

To determine if RAP1 can interact with GFAPɛ and PS1 in human cells, we used the human neuroblastoma cell line SH-SY5Y. Cell lysates were preincubated with magnetic protein G beads to eliminate any non-specifically binding proteins. Rabbit antibodies to RAP1 or non-specific rabbit antibodies crosslinked to fresh magnetic protein G beads were incubated with cell extracts. Proteins eluted from the antibodies were resolved by SDS-PAGE and detected by immunoblotting. GFAPɛ coprecipitated with RAP1, but GFAPɛ did not precipitate with the non-specific antibodies (Fig. 3, right panel). Under the conditions we used, we were unable to detect PS1 in the RAP1 precipitated samples (data not shown).

### RAP1 colocalizes with GFAPɛ and gamma secretase subunits in sh-SY5Y cells

**Fig. 4.**
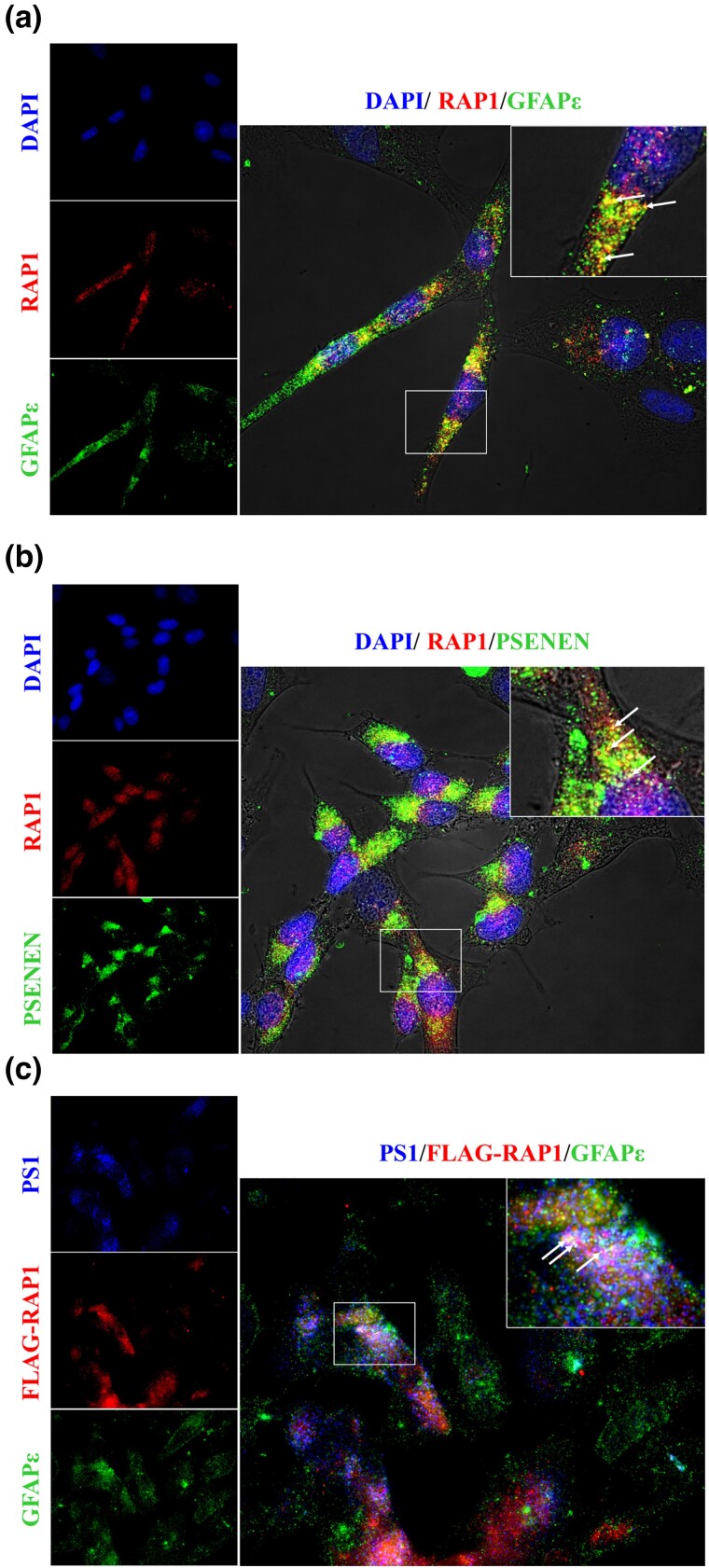
RAP1 and GFAPɛ colocalize with subunits of the γ-secretase complex. SH-SY5Y cells were grown, fixed, and probed with antibodies as indicated in each panel. Representative images are shown that include images of each fluorophore individually with a larger, merged image. The box in the merged images indicates the region that is increased in size for the inset at the top right corner. Arrows in the inset indicate examples of colocalization. a) RAP1 and GFAPɛ colocalize. Cells were probed with DAPI, antibodies to RAP1 and antibodies to GFAPɛ. b) RAP1 colocalizes with the γ-secretase subunit PSENEN. Cells were probed with DAPI, antibodies to RAP1, and antibodies to PSENEN. c) RAP1 and GFAPɛ colocalize with the γ-secretase subunit PS1. DAPI stain was not used in this image. Cells were probed with antibodies to PS1, antibodies to RAP1 and antibodies to GFAPɛ.

To determine if RAP1, GFAPɛ and PS1 are found together in human cells, we used immunofluorescence with fixed SH-SY5Y cells to localize these proteins. Figure 4a shows that endogenous RAP1 (red) and endogenous GFAPɛ (green) colocalized (yellow color in the overlay panel), though each protein was also found independently of the other. In Fig. 4b, endogenous RAP1 (red) and the γ-secretase subunit PSENEN (green) also colocalized (yellow color in the overlay panel) and were also found independently of one another. To determine if all three proteins colocalize simultaneously in the cells, we expressed a FLAG-tagged RAP1 protein to include antibodies to the PS1 subunit of γ-secretase. The data in Fig. 4c indicate that all three proteins colocalized (white in the overlay panel).

### RAP1 and GFAPɛ alter gamma secretase activity in a reconstituted yeast system

**Fig. 5.**
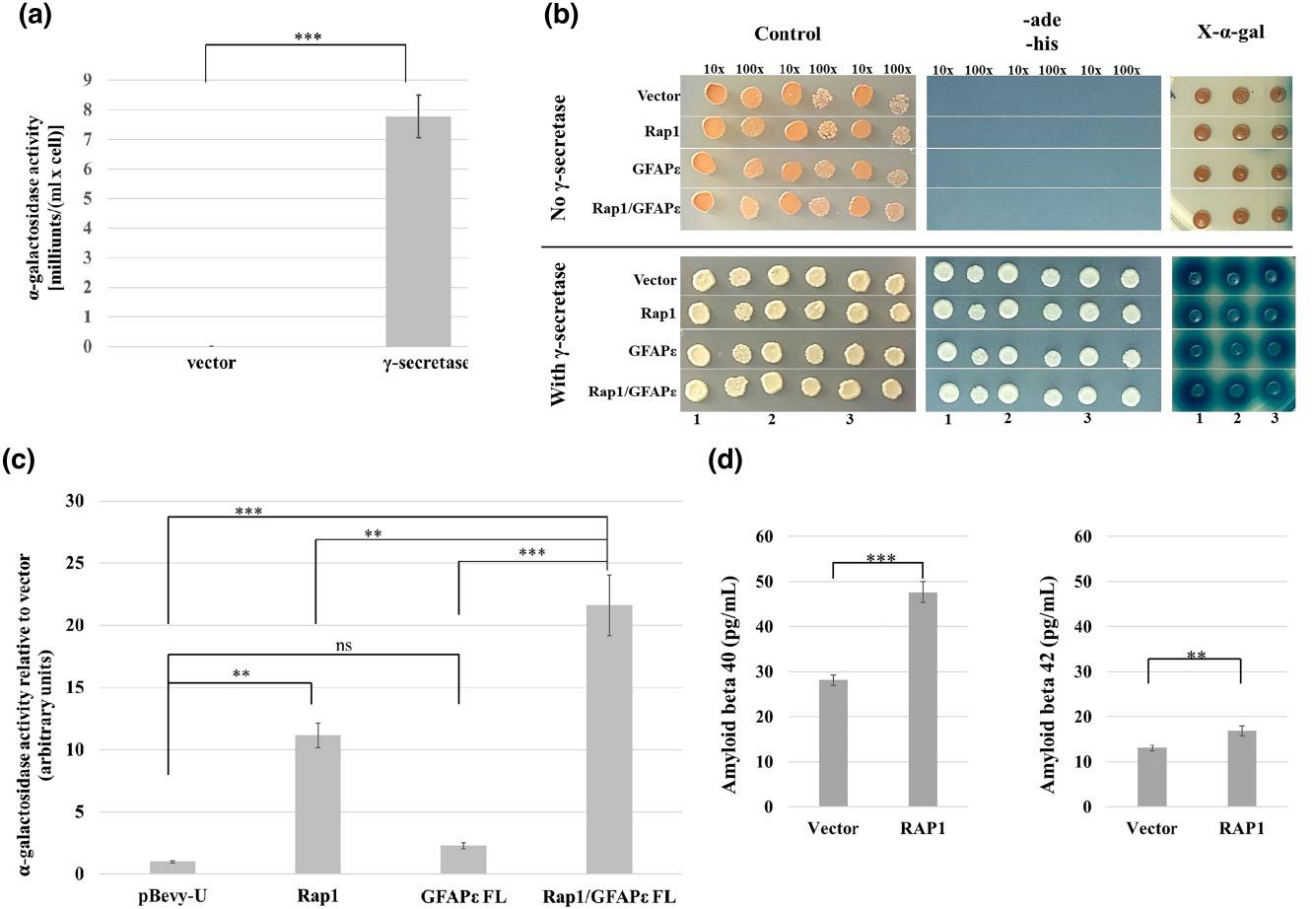
RAP1 and GFAPɛ affect the activity of human γ-secretase expressed in yeast. a) Strain MY14 (expressing C99-Gal4p) was transformed with pBEVY-T and pBEVY-L vectors or the pBEVY plasmids expressing the four subunits of the γ-secretase complex. Four independent transformants of each were grown for use in an α-galactosidase activity assay. The bars on the graph represent the average α-galactosidase activity with error bars representing the standard error of the mean. The *P*-value was determined using a one-tailed student's *t*-test (***P < 0.001). b) Strain MY14 (expressing C99-Gal4p) with pBEVY-T and pBEVY-L vectors or the pBEVY plasmids expressing the four subunits of the γ-secretase complex were transformed with the pBEVY-U vector, or pBEVY-U expressing RAP1, GFAPɛ, or both RAP1 and GFAPɛ. Three independent transformants of each were assayed. Cells were grown in SC-trp-leu-ura medium. Cells were spotted directly onto SC-trp-leu-ura medium with X-α-gal (panels on right) or diluted before plating the 1:10 and 1:100 dilutions on SC-trp-leu-ura (control) medium (left panels) or SC-trp-leu-ura-ade-his medium (center panels). The dilutions are indicated above the images, and the sample numbers are indicated at the bottom. c) Strain MY14 (expressing C99-Gal4p) with the pBEVY plasmids expressing the four subunits of γ-secretase were transformed with the pBEVY-U vector, or pBEVY-U expressing RAP1, GFAPɛ, or both RAP1 and GFAPɛ. Three independent transformants of each were used, and three technical replicates for each assayed for α-galactosidase activity. The technical replicate values were averaged to represent each of the biological replicates. The bars on the graph represent the average α-galactosidase activity of the biological replicates relative to the average activity of the control for simplicity. Error bars represent the standard error of the mean of the biological replicates. The *P*-values were determined using a nested, one-way ANOVA with a Tukey's multiple comparisons test (**P < 0.01; ***P < 0.001; ns, not significant). d) U251 cells were transfected with pLPC vector or pLPC-hRAP1 FL that overexpresses RAP1. Six wells transfected with each plasmid were used to measure Aβ40 and Aβ42. Two technical replicates for each were measured using ELISA. The bar graphs represent the average concentration of the peptides in the media. Error bars represent the standard error of the mean of the biological replicates. *P*-values were determined using a two-tailed student's *t*-test (**P < 0.01; ***P < 0.001).

The interactions and colocalization among RAP1, GFAPɛ, and subunits of the γ-secretase complex suggest that RAP1 and/or GFAPɛ may affect γ-secretase activity. We tested the effects of RAP1 and GFAPɛ using a model of γ-secretase activity in *S. cerevisiae*, similar to previous reports (Edbauer *et al.* 2003; Futai *et al.* 2009). We cloned the genes encoding the four subunits of human γ-secretase (*APH1A, NCSTN*, *PSENEN*, and *PSEN1*). We used PS1 (encoded by *PSEN1*) as the catalytic subunit instead of PS2 (encoded by *PSEN2*) since most of the known mutations leading to EOAD occur in the *PSEN1* gene (Cruts *et al.* 2012). Our reporter gene for γ-secretase activity was a fusion of C99, the product of β-secretase cleavage of APP, and the yeast Gal4p transcriptional activator. In human cells, the transmembrane C99 is cleaved into amyloid beta peptides and the AICD, and both fragments are freed from the membrane. In the yeast system, the C99 portion of the C99-Gal4p fusion protein will be inserted into the plasma membrane, sequestering Gal4p outside of the nucleus, preventing activation of genes under Gal4p control. Cleavage of C99 by γ-secretase will release Gal4p from the plasma membrane, enabling it to enter the nucleus, and activate genes under its control. We expressed the C99-Gal4p fusion protein from the chromosome by targeting it to the *MET17* locus. We used a strain with *HIS3* and *ADE2* under the control of Gal4p, allowing growth on medium lacking histidine and adenine to serve as a proxy for γ-secretase cleavage of C99. Gal4p also controls the wild-type *MEL1* gene, encoding α-galactosidase, in this strain which can be used to obtain quantitative data. The reporter strain MY14 was transformed with plasmids expressing the four γ-secretase subunits. The data in Fig. 5a show that when the γ-secretase subunits were expressed in this strain, there was a significant increase in α-galactosidase activity. Cells without γ-secretase expression did not grow on SC-trp-leu-ade-his, and the X-α-gal did not turn blue, regardless of whether RAP1 and/or GFAPɛ were expressed (Fig. 5b, top panels). When γ-secretase was expressed in MY14, the cells grew on SC-trp-leu-ade-his, and X-α-gal turned blue (Fig. 5b, bottom panels). Expression of RAP1 and/or GFAPɛ had no visible consequences on these qualitative phenotypes. Expression of GFAPɛ alone did not result in a significant change in α-galactosidase activity compared to that of cells containing just the vector (Fig. 5c). RAP1 expression resulted in a significant increase in activity. Expression of GFAPɛ in addition to RAP1 resulted in a significant increase of α-galactosidase activity over that of the cells expressing RAP1 alone. These data indicate that RAP1 and GFAPɛ can increase γ-secretase activity on C99 cleavage in the yeast system. To validate the yeast results, human U251 glioblastoma cells were transiently transfected with vector or a plasmid overexpressing RAP1, and the media were analyzed for secreted Aβ40 and Aβ42. Cells overexpressing RAP1 showed modest but significant increases in Aβ40 and Aβ42 (Fig. 5d), indicating that overexpression of RAP1 increases γ-secretase activity on APP in human cells.

## Discussion

In addition to its role in telomere protection as part of the shelterin complex (de Lange 2005), the RAP1 (TERF2IP) protein is also localized in the cytoplasm of cells where it functions in NF-kB signaling (Teo *et al*. 2010). In this study, we show that RAP1 forms a complex with GFAPɛ and PS1 in vitro, and RAP1 and GFAPɛ colocalize in human cells with γ-secretase indicating they form a complex in vivo. RAP1 increased γ-secretase activity in a reconstituted yeast system, which was validated by overexpressing RAP1 in human cells leading to increased Aβ production. Thus, RAP1 has another cytoplasmic role in the activation of γ-secretase, which may increase as cells age or in some cases of AD. As normal human fibroblasts aged, telomere length shortened and TRF2 levels declined, but RAP1 levels remained higher than expected in both the nucleus and cytoplasm, suggesting an age-related function for RAP1 (Swanson *et al*. 2016). Age is the greatest risk factor for the development of AD, including EOAD, though aging is not a direct cause of the disease. Thus, as cells age, increased extratelomeric RAP1 may lead to an increase in γ-secretase activity and increased Aβ production and deposition.

Edbauer *et al*. (2003) reconstituted human γ-secretase in yeast. The yeast model has been used to study the effects of specific γ-secretase subunit variants (Yonemura *et al*. 2011; Yonemura *et al*. 2016), the biochemical effects of PS1 mutants that cause EOAD (Futai *et al*. 2016; Imai *et al*. 2019), and other mutations, increasing our understanding of how subunits within the complex function (Edbauer *et al*. 2004; Futai *et al*. 2009; Futai *et al.* 2019; Watanabe *et al*. 2022). To our knowledge, our study is the first to investigate modulators of γ-secretase activity using this system. Using yeast, we have shown that RAP1 activates γ-secretase. GFAPɛ further potentiated the effect of RAP1. Since GFAPɛ transcripts are increased in human AD brains where higher transcript levels correlated with increased severity of the disease (Kamphuis *et al*. 2014), the synergistic effect shown by RAP1/GFAPɛ is important.

Though the increase in γ-secretase activity caused by RAP1 overexpression was modest, it is important considering AD can be caused by an increase in overall γ-secretase activity as seen in the APP_swe_ mutation (Citron *et al*. 1992; Weggen and Beher 2012) or in trisomy 21, in which there is a 50% increase in the number of *APP* genes. In these individuals, AD neuropathologies are seen by the age of 40 (Lott and Head 2019). The modest increase in Aβ production due to RAP1 overexpression is likely because RAP1 does not completely overlap with γ-secretase in vivo. RAP1 has other cytoplasmic functions. γ-Secretase has many target proteins that it cleaves, which may require different γ-secretase complexes or different accessory factors to cleave specific targets (Haapasalo and Kovacs 2011; Carroll and Li 2016). Thus, RAP1 is a novel member of a growing class of factors known as γ-secretase modulatory proteins (GSMPs), which have been shown to modify γ-secretase activity differently on APP and Notch1 (Wong *et al*. 2020). GSMPs are emerging as possible targets of therapeutics since chemical modifiers have met with little success (Luo and Li 2022).

Oxidative stress, caused by the buildup of reactive oxygen species (ROS; Balaban *et al.* 2005), and shortening of telomeres (Harley *et al.* 1992) are known components of the normal, cellular aging process. In AD brains, oxidative stress is elevated (Pratico and Sung 2004) and telomeres markedly reduced (Mahady *et al.* 2021) compared to normal samples. Oxidative stress affected the distribution of RAP1 in U251 glioblastoma cells, where nuclear RAP1 declined while cytoplasmic RAP1 persisted (Swanson *et al*. 2016). Shortening of telomeres is accelerated with DNA damage caused by ROS (Singh *et al*. 2019), which would free RAP1 from the telomere to perform other functions in the cell. Although the exact mechanism as to why our brains produce Aβ peptides is not understood, oxidative stress seems to play a major role since elevated levels of Aβ peptides have been reported with increased levels of oxidation products in AD brains (Butterfield and Lauderback 2002). Thus, RAP1 may normally function to increase γ-secretase activity in response to oxidative stress in the brain, and in certain genetic contexts, RAP1 may exacerbate or cause the development of AD. This is underscored by the fact that expression of a RAP1 mutation unable to bind TRF2, which localized to the cytoplasm, resulted in mice that had neurological deficits and exhibited astrogliosis (Stock *et al.* 2022), a characteristic feature of AD (Osborn *et al*. 2016).

The yeast system provides a simple yet powerful approach to studying γ-secretase and GSMPs. This system can easily detect changes in γ-secretase activity in a background that does not have competing pathways. Using this system, the effects of variants and mutations of RAP1, GFAPɛ, APP, and the presenilins can help show how these proteins function together in EOAD to provide insight into the mechanisms that produce Aβ peptides leading to plaque formation, a hallmark of AD pathology.

## Supplementary Material

jkad057_Supplementary_Data

## Data Availability

Strains and plasmids are available upon request. The authors affirm that all data necessary for confirming the conclusions of the article are present within the article, figures, and tables. Supplemental material available at G3 online.
